# Use of Real-World Evidence in Health Technology Assessments of Non-Oncology Rare Disease Therapies

**DOI:** 10.3390/jmahp14020032

**Published:** 2026-05-21

**Authors:** Oliver Blandy, Pierluigi Lembo, Rebecca Folorunso, Karl-Johan Myren, Helene Chevrou-Severac, Simu K. Thomas

**Affiliations:** 1 Clarivate, 70 St Mary Axe, London EC3A 8BE, UK; 2 Health Economics and Outcomes Research, Alexion, AstraZeneca Rare Disease, Forskaren, Hagaplan 4, 11368 Stockholm, Sweden; 3 Health Economics and Outcomes Research, Alexion, AstraZeneca Rare Disease, Neuhofstrasse 34, 6340 Baar, Switzerland; 4 Health Economics and Outcomes Research, Alexion, AstraZeneca Rare Disease, 121 Seaport Blvd, Boston, MA 02210, USA

**Keywords:** real-world evidence, real-world data, non-oncology orphan medicinal products, rare disease, health technology assessment, orphan drug, orphan medicine

## Abstract

**Background:** Real-world evidence (RWE) can complement clinical trials to inform health technology assessments (HTAs). This study examined the extent to which RWE is considered in HTAs of non-oncology orphan medicinal products across six agencies globally. **Methods:** Published European Medicines Agency decisions were reviewed to identify approved non-oncology orphan medicinal products (2018–2023) that included RWE within their submission package, which was anticipated to align with the inclusion of RWE in HTA submissions. Data were extracted from the corresponding HTA reports published by six national agencies (Australia, Canada, France, Germany, Sweden, and the UK). **Results:** RWE was included in 105 regulatory submissions and 52.6% of the corresponding HTA reports (range: 29.9% [Germany] to 78.8% [Canada]), nearly 90% of which received a positive decision (range: 44.4% [Australia] to 100.0% [Germany]). RWE was derived from a variety of study designs and commonly supported clinical efficacy across many therapeutic areas. **Conclusions:** RWE commonly supports HTAs of recently approved non-oncology orphan medicinal products, strengthening the evidence base and contributing to demonstration of product value.

## 1. Introduction

Rare diseases are increasingly gaining recognition as a global health priority, with 95% of these conditions lacking approved treatments [1] and affected patients (approximately 300 million people worldwide) commonly experiencing substantial unmet health needs [2]. For rare disease therapies, conducting extensive randomized controlled trials (RCTs) may not be feasible due to limited patient numbers, ethical considerations associated with administering a placebo to patients with a severe disease, and the complexity of trial execution, all of which challenge the production of robust supporting clinical evidence [3]. Although supporting evidence for rare disease treatments often comes from small or short-term RCTs or, in some cases, single-arm studies, real-world evidence (RWE) can also be utilized [3].

RWE, such as historical control arms, can be used to fill evidence gaps and to strengthen the evidence base for rare disease therapies, thus facilitating regulatory approval, reimbursement, and patient access [3,4]. Regulatory agencies may grant conditional approval of rare disease treatments while additional data is gathered to confirm clinical benefits [4,5]. For novel treatments, including those for rare diseases, RWE can be used to demonstrate long-term effectiveness and safety in broader patient groups than those participating in clinical trials, as well as to identify patterns in medication use and adherence in real-world settings [6,7,8]. Patients with rare diseases have also been shown to have disproportionately higher healthcare resource utilization and cost compared with those diagnosed with more common conditions [9], and RWE can be used to quantify the disease burden for patients (e.g., their perceptions of disease management), caregivers, and the healthcare system [10].

RWE is frequently used to support regulatory applications; an analysis of European Medicines Agency (EMA) resources showed that approximately 40% of all marketing authorization applications and extensions of indication submitted in 2018 and 2019 included RWE [11]. Guidance has been published on the use of RWE in both regulatory decision-making [12] and in health technology assessment (HTA) [13,14,15,16], with studies reporting growing use of RWE in HTA submissions over time [17,18]. However, a recent study of HTA agencies found that, while most agencies generally consider RWE within their guidelines, the degree to which it contributes to decision-making differs [19]. Although some HTA agencies encourage the use of RWE as a complement to RCT data, others only consider it in the absence of RCT data [19]. Moreover, several HTA agencies only provide limited detail or guidance, meaning there is still a lack of clarity about the type of RWE that can be used in HTA submissions, and under which circumstances it can be utilized [19].

While some studies have examined the use of RWE in HTA submissions for oncology medicines [20] or rare disease therapies as a whole [18], there is a paucity of studies that focus on the role of RWE in the assessment of non-oncology orphan medicinal products (OMPs). To fill this gap, the objective of our study was to understand the extent to which RWE is considered as part of value assessments of OMPs across six HTA bodies globally, and the extent to which reports that include RWE receive positive decisions.

## 2. Materials and Methods

The database of published EMA decisions from the Committee for Medicinal Products for Human use (CHMP) was reviewed to identify OMPs approved within the last 5 years (2018–2023) that included RWE as part of their submission package. This review of the EMA database was conducted as it was assumed that the inclusion of RWE in EMA-approved regulatory submissions would align with the inclusion of RWE in HTA submissions. Manual searches of the EMA website were conducted by a single reviewer to identify OMPs approved within the specified time frame. Identified submission packages were screened based on title/summary to exclude oncology OMPs. Full texts of potentially relevant reports were then examined by a single reviewer to identify the inclusion of RWE; selected reports were independently verified by a second reviewer (any disputes around eligibility for inclusion in the analysis were referred to a third reviewer). Key terms used to identify possible inclusion of RWE were: ‘retrospective’, ‘registry’, ‘observational’, ‘historical’, ‘real’, ‘claims’, ‘external’, ‘survey’, ‘electronic’, ‘real-world’, ‘real world’, ‘administrative’, ‘non interventional’, ‘non-interventional’, ‘world’, ‘network meta analysis’, ‘network meta-analysis’, ‘NMA’, ‘RWD’, and ‘RWE’. The list of EMA-approved OMPs with a positive indication of RWE use in their submission packages was extracted into a data extraction table in Microsoft Excel.

For the identified OMPs, we conducted an analysis of the HTAs performed by six national agencies: Canada’s Drug Agency (CDA-AMC, formerly known as the Canadian Agency for Drugs and Technology in Health) (Canada), Haute Autorité de Santé (HAS) (France), Institut für Qualität und Wirtschaftlichkeit im Gesundheitswesen (IQWiG) (Germany), National Institute for Health and Care Excellence (NICE) (England), Pharmaceutical Benefits Advisory Committee (PBAC) (Australia), and Tandvårds och läkemedelsförmånsverket (TLV) (Sweden). These agencies were selected to cover a spectrum of key European and global bodies. For Germany, only HTA reports of OMPs that underwent a full IQWiG assessment were included in the analysis, which takes place after the sales exceed €30 million (€50 million in the past [21]). Each HTA agency website was hand-searched by a single reviewer to locate relevant HTA reports for the identified OMPs. Findings were recorded in a data extraction table; extracted HTA reports were verified by a second reviewer, with any disputes referred to a third reviewer. Most HTA reports were available in English, but for those that were not, Google Translate was used for translation. If a HTA agency did not have a corresponding report for an identified EMA-approved OMP, the reasons behind this lack of data were not determined.

Each HTA report was individually examined to determine whether RWE was included as part of the evidence package, as well as the outcome of the assessment (i.e., a positive or negative decision). For each HTA report with RWE, data describing the study design(s) used to gather RWD, the specific section(s) of the HTA report supported by RWE (i.e., clinical efficacy, safety, quality of life [QoL], and economic evidence), and therapeutic areas were extracted and analyzed using descriptive statistics. The results are presented for the overall data set and for each HTA body included in the analysis.

## 3. Results

**Figure 1:**
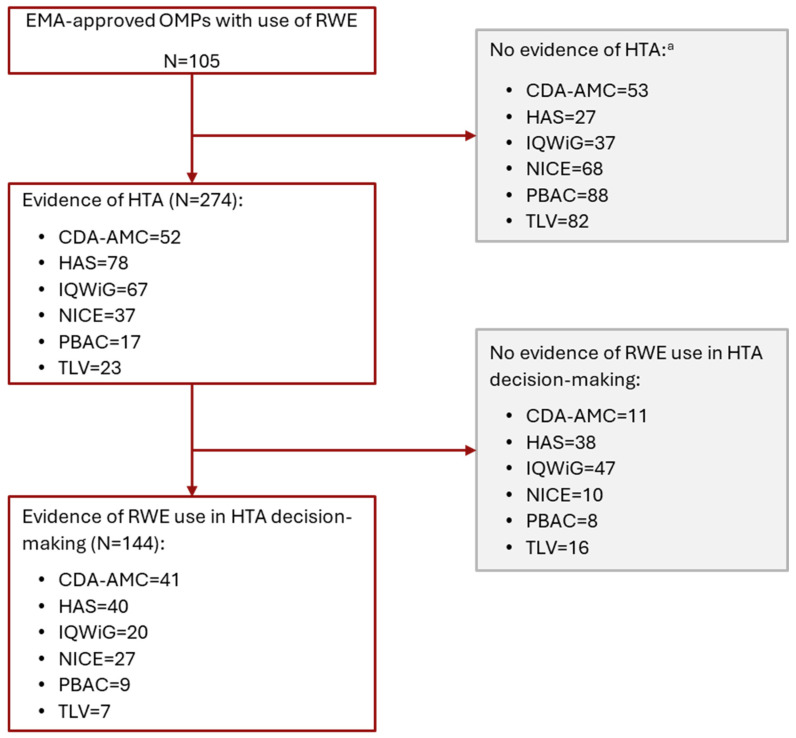
The inclusion of RWE in EMA and HTA submissions for OMPs. a: No HTA report was found for the EMA-approved OMP. Companies may have chosen not to submit an appraisal, or the appraisal was either terminated or is still in the process of development. The reasons behind this lack of data were not verified in our analysis. Abbreviations: CDA-AMC, Canada’s Drug Agency; EMA, European Medicines Agency; HAS, Haute Autorité de Santé; HTA, health technology assessment; IQWiG, Institut für Qualität und Wirtschaftlichkeit im Gesundheitswesen; NICE, National Institute for Health and Care Excellence; OMP, non-oncology orphan medicinal product; PBAC, Pharmaceutical Benefits Advisory Committee; RWE, real-world evidence; and TLV, Tandvårds och läkemedelsförmånsverket.

Between 2018 and 2023, 105 OMPs received EMA regulatory approval and included RWE in their EMA submission as supporting data. Across the six HTA agencies included in this study, there were 274 HTA reports associated with these EMA-approved OMPs (HAS: 78; IQWiG: 67; CDA-AMC: 52; NICE: 37; TLV: 23; PBAC: 17 [Figure 1]). The list of all reviewed EMA approvals and the corresponding HTA reports is provided in the Supplementary Materials.

**Figure 2:**
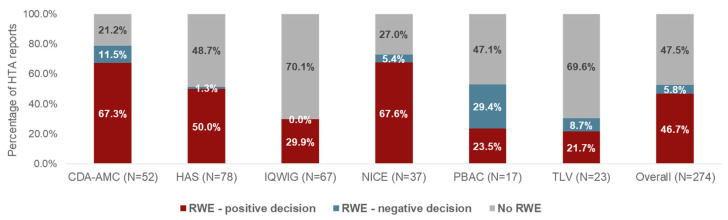
HTA reports which included RWE and the resultant decisions, by HTA agency. Abbreviations: CDA-AMC, Canada’s Drug Agency; HAS, Haute Autorité de Santé; HTA, health technology assessment; IQWiG, Institut für Qualität und Wirtschaftlichkeit im Gesundheitswesen; NICE, National Institute for Health and Care Excellence; OMP, non-oncology orphan medicinal product; PBAC, Pharmaceutical Benefits Advisory Committee; RWE, real-world evidence; and TLV, Tandvårds och läkemedelsförmånsverket.

As illustrated in Figure 2, 144 of the 274 HTA reports (52.6%) included RWE, with the highest RWE utilization in submissions filed in Canada and England (CDA-AMC: 78.8% [*n* = 41/52]; NICE: 73.0% [*n* = 27/37]; PBAC: 52.9% [*n* = 9/17]; HAS: 51.3% [*n* = 40/78]; TLV: 30.4% [*n* = 7/23]; and IQWiG: 29.9% [*n* = 20/67]). The remaining 47.4% of HTA reports did not contain RWE, despite RWE being included in the corresponding EMA submissions.

Of the 144 submissions that included RWE, 128 (88.9%) were granted a positive HTA decision; the highest proportion of reports with RWE that were granted a positive HTA decision was observed for IQWiG (100%, *n* = 20/20), followed by HAS (97.5%, *n* = 39/40), and NICE (92.6%, *n* = 25/27) (Figure 2).

**Figure 3:**
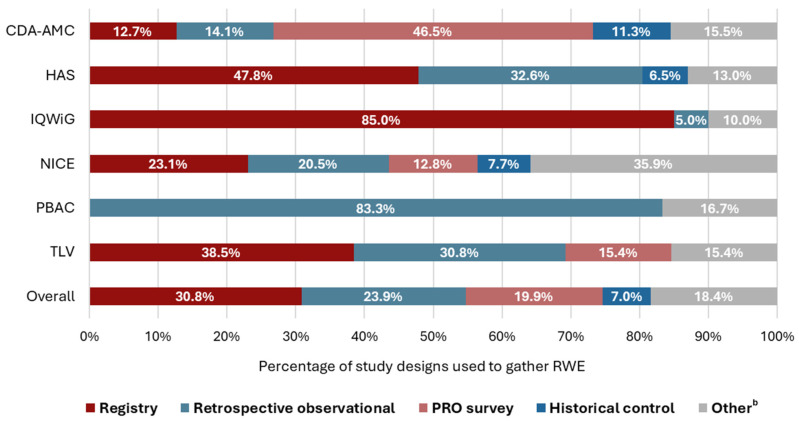
Study designs for RWE included in assessment reports, by HTA agency ^a^. a: Multiple studies, and therefore multiple study designs, may have been used to gather the RWE included in a HTA report. Overall, 201 RWD studies were included across 144 HTA reports. b: Included non-interventional, retrospective cohort, healthcare practitioner survey, natural history, prospective observational, electronic medical record, indirect treatment comparison, systematic literature review/network meta-analysis, administrative database analysis, claims, patient preference, and prospective cohort studies. Abbreviations: CDA-AMC, Canada’s Drug Agency; HAS, Haute Autorité de Santé; HTA, health technology assessment; IQWiG, Institut für Qualität und Wirtschaftlichkeit im Gesundheitswesen; NICE, National Institute for Health and Care Excellence; OMP, non-oncology orphan medicinal product; PBAC, Pharmaceutical Benefits Advisory Committee; PRO, patient-reported outcome; RWE, real-world evidence; and TLV, Tandvårds och läkemedelsförmånsverket.

Overall, 201 RWD studies were included across 144 HTA reports. The analysis of the HTA reports including RWE demonstrated that the most common study designs were registries (*n* = 62/201 [30.8%]), followed by retrospective observational studies (*n* = 48/201 [23.9%]), and patient-reported outcome (PRO) surveys (*n* = 40/201 [19.9%]) (Figure 3). However, the utilization of different study designs varied substantially by agency; for example, no historical control studies were considered by the IQWiG, PBAC, or TLV.

**Figure 4:**
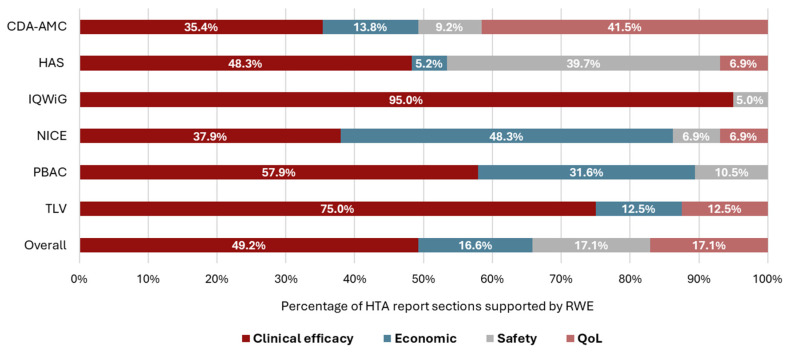
Sections of the HTA reports supported by RWE, by HTA agency ^a^. a: More than one section of each HTA report may have been supported by RWE. Overall, RWE was used to support 199 different clinical efficacy, economic, safety, or QoL sections across 144 HTA reports. Abbreviations: CDA-AMC, Canada’s Drug Agency; HAS, Haute Autorité de Santé; HTA, health technology assessment; IQWiG, Institut für Qualität und Wirtschaftlichkeit im Gesundheitswesen; NICE, National Institute for Health and Care Excellence; OMP, non-oncology orphan medicinal product; PBAC, Pharmaceutical Benefits Advisory Committee; QoL, quality of life; RWE, real-world evidence; and TLV, Tandvårds och läkemedelsförmånsverket.

Overall, RWE was used to support 199 different clinical efficacy, economic, safety, or QoL sections across 144 HTA reports. The most commonly supported sections were clinical efficacy (*n* = 98/199 [49.2%]), followed by safety (*n* = 34/199 [17.1%]), QoL (*n* = 34/199 [17.1%]), and economic value (*n* = 33/199 [16.6%]) (Figure 4).

**Figure 5:**
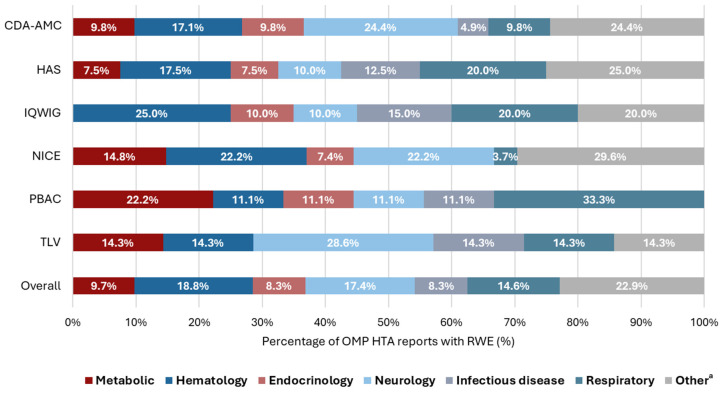
The therapeutic areas for which RWE was considered, by HTA body. a: ‘Other’ therapeutic areas (<5% of HTA reports with RWE) included immunology, rheumatology, cardiology, hepatic, dermatology, nephrology, gastrointestinal, and multiple therapeutic areas. Abbreviations: CDA-AMC, Canada’s Drug Agency; HAS, Haute Autorité de Santé; HTA, health technology assessment; IQWiG, Institut für Qualität und Wirtschaftlichkeit im Gesundheitswesen; NICE, National Institute for Health and Care Excellence; OMP, non-oncology orphan medicinal products; PBAC, Pharmaceutical Benefits Advisory Committee; RWE, real-world evidence; and TLV, Tandvårds och läkemedelsförmånsverket.

The HTA reports supported by RWE (*n* = 144) covered a wide range of therapeutic areas, with the most common being hematology (*n* = 27/144 [18.8%]), neurology (*n* = 25/144 [17.4%]), and respiratory (*n* = 21/144 [14.6%]) diseases (Figure 5).

## 4. Discussion

We found that HTA bodies from around the world (CDA-AMC, HAS, IQWiG, NICE, PBAC, and TLV) are likely to consider RWE as part of submission packages for OMPs. Among OMPs citing RWE in the EMA dossiers, approximately half of the corresponding HTA reports also included RWE. However, the inclusion of such evidence varied considerably between HTA agencies, ranging from 29.9% for IQWiG to 78.8% for CDA-AMC. Overall, nearly 90% of HTA submissions that included RWE received a positive decision. Among individual agencies, the proportion of positive decisions for RWE-containing submissions ranged from 100% for IQWiG to 44.4% for PBAC.

The between-agency variation is potentially due to differences in HTA guidelines. While NICE, HAS, and CDA-AMC offer explicit guidance on RWE use in HTA, TLV has only published limited guidance that focuses on the application of RWE in oncology [19]. Unlike IQWiG, which notes a preference for RCTs and consideration of RWE only when RCT data is unavailable, PBAC makes no mention of RWE in relation to clinical evidence in their guidelines [19]. Generally, although major HTA agencies have started to encourage the inclusion of RWE in HTA submissions in their methods and guidance [3], guidelines often provide limited detail [19].

In our study, RWE was most commonly used to support clinical efficacy from RCTs. Notably for IQWiG submissions, RWE was only used to support the efficacy and safety sections. This is aligned with the exclusion of cost-effectiveness analysis in the German HTA process, in which product value is assessed primarily in relation to the clinical benefits for patients [22,23]. Examples of submissions, in which the use of RWE to support RCT data in the efficacy and safety sections was prominent, include NICE TA988, a reassessment of CFTR modulators for the treatment of cystic fibrosis [24]. The initial appraisal of lumacaftor–ivacaftor established a comprehensive data collection agreement utilizing the UK Cystic Fibrosis Registry and several additional data sources, and the collected RWE was subsequently used alongside the data from RCTs and open-label extension studies to support long-term clinical effectiveness (as well as inform quality of life benefits associated with CFTR modulators and the economic model) [24]. Similarly, NICE HST23, a reassessment of asfotase alfa for treating pediatric-onset hypophosphatasia, used data for all patients treated with asfotase alfa in the National Health Service, which were collected as part of a managed access agreement implemented following the initial appraisal [25]. This data, alongside RWE from a global registry, was instrumental in confirming the efficacy of asfotase alfa in patients with severe juvenile-onset hypophosphatasia, supplementing information from RCTs and extension studies to enable access to treatment for a broader population of patients [25].

Less frequently, RWE has been used as the primary source of data supporting clinical efficacy in the HTA setting. The clinical efficacy of nusinersen in adults with type II or III spinal muscular atrophy was supported solely by RWE, and the Canadian Agency for Drugs and Technologies in Health reached a negative decision, citing as a rationale the limitations of observational studies and the resulting inconclusive evidence on efficacy [26]. Although oncology indications were outside of the scope of this review, a similar example is the use of RWE is lutetium (^177^Lu) oxodotreotide in treating neuroendocrine tumors. Clinical efficacy was initially determined based real-world data collected by the Erasmus Medical Center in the Netherlands [27]. This preceded collection of RCT evidence through the NETTER-1 trial [28], and both sources of data were considered in the NICE appraisal of this product [29].

Beyond clinical efficacy and safety, RWE supported economic sections (including cost-effectiveness models extrapolating the long-term effectiveness of the OMP using RWE) and QoL sections, especially for appraisals submitted to NICE and CDA-AMC, in which these uses of RWE predominated. This likely reflects both agencies basing the assessment of product value on cost-effectiveness analysis, which makes economic value (including the cost-effectiveness models extrapolating long-term effectiveness and the needed QoL data inputs) a key part of the HTA [22,30,31]. For example, in a recent gene therapy appraisal in sickle cell disease, RWE from a burden of disease study and a survey were used to contextualize the disease and its treatment. These data raised important equity issues such as socioeconomic deprivation and stigmatization that may prevent patients from seeking and receiving appropriate care [32].

The findings from our analysis are in line with those in the available literature. A systematic review conducted by Aggarwal et al. (2022) found that, for ~90% (*n* = 17/19) of ultra-rare disease treatments assessed under NICE’s highly specialized technology (HST) program (2016–2022), the use of RWE was requested, considered, or accepted [33]. As in our analysis, a variety of study designs were used to derive the RWE included in the HST reports, but most were from registry studies (42%) [33]. Another study also reported that the use of RWE in HTA submissions varies by HTA agency; in an analysis of 130 appraisals for rare disease medicines from CDA-AMC and NICE (2009–2019), 67% and 37% of the submissions to CDA-AMC and NICE included RWD, respectively [18]. Similarly, the key role of RWE in supporting the HTA submissions specifically in rare diseases clearly emerged from a recent review of RWE use in HTA reassessments across six agencies worldwide. More than three quarters of HTAs using RWE were for orphan therapies and, conversely, orphan drugs contributed only 22% of HTAs that did not use RWE [34]. The challenges surrounding HTA of orphan drugs are well understood [35]. The availability of data from large RCTs utilizing well-established endpoints is limited in rare diseases and the increased use of RWE, informed by appropriate guidance from HTA agencies, could help mitigate uncertainty in HTAs of OMPs [35].

Increased use of RWE could enable a more complete demonstration of product value and facilitate decision-making in HTA. RWE provides country-specific data that is not available from RCTs, including information on local treatment pathways and disease management, healthcare resource utilization, and costs. It can also describe the patient perspective in more depth than structured assessments of QoL utilized in RCTs. In the era of personalized medicine, RWE may provide important data on the effectiveness and safety of an OMP in patients with uncommon disease types or other characteristics that would make them under-represented in RCTs conducted in an already rare disease (e.g., patients with a non-HbSS genotype in trials of gene therapies in sickle cell disease [36,37]). RWE can also provide an external control for RCT data, which is particularly valuable in conditions in which the use of placebo would be unethical (as seen, e.g., in NICE HST23 in hypophosphatasia [25]). Finally, the use of RWE in health technology reassessment can help to verify the initial decision of the agency and potentially lead to broader access to treatment following reassessment (as in NICE HST23 [25]). Technological advancements will likely improve quality and acceptability of RWE, as the availability of electronic data is increasing and the analytical tools are improving, allowing researchers to tap into previously difficult to analyze data, such as free-text fields in registries. To fully harness the potential benefits of RWE for HTA, there is a need for consistent guidance on RWE use across HTA agencies and a consistent attitude towards the resulting data. This is particularly crucial in Europe in the setting of the Joint Clinical Assessment (introduced by Regulation (EU) 2021/2282) that will be extended to all OMPs in 2028 [38], providing an opportunity for the harmonization of RWE acceptance across the European agencies, but also creating an urgent need for joint guidance on RWE use in HTA across Europe.

The design of this study and the presented results have some limitations. It should be noted that, by using the EMA database to identify approved OMPs for inclusion in our analysis, we may have missed OMPs without EMA approval but with regulatory approval in Australia or Canada. Oncology indications, in which the use of RWE in HTA is relatively well-established, were not included in the analysis. It is likely that their inclusion could have increased the proportion of HTA reports that utilize RWE. Additionally, the findings of this study rely on the transparency of each HTA agency and the information that is made public, both of which vary from agency to agency [39,40]. The comparison of RWE use in submission packages across agencies is limited by the low number of assessment reports identified for some of the HTA bodies. Furthermore, if a HTA agency did not have a corresponding report for an identified EMA-approved OMP, it was outside the scope of this study to determine the reasons behind this. It was assumed that the company may have chosen not to submit an appraisal or that the appraisal may have been in process or terminated. Notably, in Germany, the IQWiG performs a full assessment of an orphan drug only after the product’s sales exceed €30 million per year (previously €50 million per year) [21,41]. Therefore, it is expected that not all EMA-approved OMPs would have an associated IQWiG report. Nonetheless, for comparability with other HTA agencies included in the analysis, the full IQWiG assessments were included, as opposed to the assessments performed directly by the Federal Joint Committee (Gemeinsamer Bundesausschuss (G-BA), which are subject to separate regulations. Similarly, the high proportion of positive HTA decisions among OMPs considered for Germany likely reflects the relevant regulations considering that these products by definition provide an additional benefit, which may be major, considerable, minor, or non-quantifiable [41,42]. Finally, while extracting data directly from HTA dossiers provides valuable insight into how and how often RWE is used in submissions to HTA bodies, it was not possible to establish whether RWE was a determinant factor for final HTA decisions. Therefore, further qualitative research is required to investigate this in more depth.

## 5. Conclusions

RWE plays an important role in supporting the value of OMPs in HTAs across a wide range of therapeutic areas, although its utilization and the proportion of positive decisions on submissions including RWE vary by agency. With technological advancements, RWE is likely to be of ever-increasing value to HTA, particularly in rare diseases, where patients often face substantial unmet health needs and conducting traditional, large clinical development programs remains a challenge. A collaborative approach amongst HTA bodies, providing clear guidance on RWE use across multiple agencies, can enhance the demonstration of product value through RWE, and can facilitate decision-making, ultimately improving access to potentially life-saving treatments for patients affected by rare diseases.

## Data Availability

The original data sets analyzed in this study are publicly available on their respective websites: EMA: https://www.ema.europa.eu/en/homepage (accessed on 20 November 2025). CDA-AMC: https://www.cda-amc.ca/ (accessed on 20 November 2025). HAS: https://www.has-sante.fr/ (accessed on 20 November 2025). IQWiG: https://www.iqwig.de/en/ (accessed on 20 November 2025). NICE: https://www.nice.org.uk/ (accessed on 20 November 2025). PBAC: https://pbac.pbs.gov.au/ (accessed on 20 November 2025). TLV: https://www.tlv.se/in-english.html (accessed on 20 November 2025).

## Supplementary Materials

The following supporting information can be downloaded at: https://www.mdpi.com/article/10.3390/jmahp14020032/s1, Supplementary File S1: Full list of reviewed European Medicines Agency approvals.
